# Alleviation of Ulcerative Colitis in Mice by Individual Fermentation of *Periplaneta americana* Powder with *L. bulgaricus* SN22 and *S. thermophilus* SN05

**DOI:** 10.3390/microorganisms14020301

**Published:** 2026-01-27

**Authors:** Qingqing Zhang, Cheng Chen, Xiaoqin Mu, Zihan Zhang, Cuiling Luo, Chenjuan Zeng, Bisong Yue, Zhenxin Fan, Lianming Du

**Affiliations:** 1 Institute for Advanced Study, Chengdu University, Chengdu 610106, China; zqingqing0822@163.com; 2 The Conservation of Endangered Wildlife Key Laboratory of Sichuan Province, College of Life Sciences, Sichuan University, Chengdu 610065, China; mmxq0223@163.com (X.M.); zhangzihan030105@163.com (Z.Z.); luocuiling2019@163.com (C.L.); bsyue@scu.edu.cn (B.Y.); 3 Wound Care Center, West China Hospital, West China School of Nursing, Sichuan University, Chengdu 610041, China; 19802851025@163.com; 4 Periplaneta Americana and Innovative Drugs Key Laboratory of Sichuan Province, Chengdu 610051, China; zengchenjuan518@163.com

**Keywords:** yogurt strains, fermentation, *Periplaneta americana* powder, ulcerative colitis, metabolomics, anti-inflammatory activity

## Abstract

The escalating global incidence of ulcerative colitis (UC) underscores the demand for novel therapeutic strategies. This study investigated the fermentation of *Periplaneta americana* (PA) powder using two conventional dairy starter strains, *Lactobacillus delbrueckii* subsp. *bulgaricus* SN22 and *Streptococcus thermophilus* SN05, to enhance its functional properties, particularly anti-inflammatory activity, via microbial processing. Both strains demonstrated favourable safety and antimicrobial activity. Untargeted metabolomics revealed that fermentation significantly altered the metabolite profile of the PA supernatant, enriching compounds with potential bioactivities, notably anti-inflammatory (e.g., 3-anisic acid) and antioxidant (e.g., vitamin U) properties. In the DSS-induced mouse colitis model, treatment with the fermented supernatant alleviated intestinal inflammation compared to the unfermented group. This was demonstrated by significantly reduced levels of the pro-inflammatory cytokines IL-1β and TNF-α, along with improved maintenance of intestinal barrier integrity. Further in vitro assays showed that the fermented supernatant significantly suppressed proliferation and clonogenicity in human HT-29 colon cancer cells, while also inducing reactive oxygen species accumulation and apoptosis. Results demonstrate these strains are multifunctional starters possessing superior antimicrobial and anti-inflammatory efficacy. This study employed LAB fermentation of insect-derived matrices to derive bioactive components. The fermentation products exhibited anti-inflammatory potential, offering a potential microbial transformation strategy for developing functional products for adjunctive UC intervention.

## 1. Introduction

Ulcerative colitis (UC), a chronic inflammatory bowel disease (IBD) primarily characterized by hematochezia and diarrhea, exhibits a globally increasing incidence [1,2,3,4]. Current pharmacological therapies are limited by unpredictable efficacy, significant side effects, and high relapse rates [5,6]. These limitations underscore the urgent need for safe, effective, and preventive intervention strategies, particularly adjunctive/alternative approaches based on functional foods. Natural products, owing to their wide availability, diverse biological activities, and generally favorable safety profiles, demonstrate significant potential for the prevention and mitigation of UC and have garnered significant attention from both the scientific community and the food industry [7,8,9].

Fermentation with lactic acid bacteria (LAB) has been widely demonstrated as an effective biotransformation strategy. This process converts natural products into novel bioactive metabolites, thereby enhancing their therapeutic efficacy, reducing potential toxicity, and generating components with improved bioavailability [10,11,12]. Numerous studies have documented successful cases where LAB fermentation of plant-derived natural products (such as Ginseng, *Astragalus membranaceus*, and *Artemisia argyi*) significantly enhanced their anti-inflammatory, antioxidant, and gut-protective functions [13,14,15]. The application of LAB fermentation technology to process *Periplaneta americana* (PA) powder is primarily aimed at achieving the following two objectives: First, to achieve function-oriented biotransformation, i.e., utilizing the metabolic activities of LAB to convert macromolecules naturally present in PA powder (such as proteins, chitin, etc.)—which may not be readily utilizable directly—into a more diverse array of small-molecule metabolites (such as short peptides, organic acids, etc.) with higher bioavailability or stronger bioactivity, and potentially generating novel beneficial metabolites, thereby enhancing its anti-inflammatory, antioxidant, and potential gut-protective functions. Second, to optimize the application potential of the raw material, i.e., potentially reducing or eliminating certain latent allergens or weakly toxic components in the PA raw material through the fermentation process, thereby improving its safety as a functional ingredient. As a class of insect-derived natural products rich in diverse components, PA powder constitutes a complex chemical system. Its macronutrients are primarily carbohydrates, crude protein, and crude fat. The protein fraction is rich in essential amino acids such as histidine, leucine, and lysine, and contains various bioactive peptides, including antimicrobial peptides and immunomodulatory peptides. The structural polysaccharide chitin accounts for approximately 15–20% of the defatted residue, and its derivatives exhibit both prebiotic and immunostimulatory activities. The lipid fraction has a high content of unsaturated fatty acids, predominantly oleic acid and linoleic acid. The mineral profile reveals high concentrations of calcium, magnesium, and iron, along with macro and trace elements such as potassium, phosphorus, and sodium. Furthermore, the powder contains nucleosides like inosine and guanosine, as well as various bioactive metabolites known for their antioxidant, antimicrobial, and cytoprotective functions, such as uric acid, allantoin, phenolic acids, sterols, and trace indoles [16,17,18]. However, research utilizing LAB fermentation for the in-depth development of this compositionally complex insect-derived natural product to enhance its functional value and safety application potential remains relatively scarce.

In China, PA has been historically employed as a traditional natural product for several centuries. It is commonly used to treat multiple disorders, including inflammation, burns, and gastrointestinal diseases [19]. Studies have demonstrated that PA and its extracts exert significant therapeutic effects on UC in animal models, primarily by modulating the gut microbiota, repairing the intestinal mucosal barrier, and suppressing inflammatory responses [20,21,22,23]. Nevertheless, PA powder in its unprocessed form exhibits inherent limitations, such as poor digestibility and absorption of some components and limited bioavailability of active constituents. To overcome these limitations and maximize its health benefits, a LAB fermentation strategy offers a promising solution. Cross-species fermentation, which utilizes microorganisms with food-grade safety (e.g., *Lactobacillus delbrueckii* subsp. *bulgaricus* (*L. bulgaricus*) [24] and *Streptococcus thermophilus* (*S. thermophilus*) [25,26]) to ferment insect-derived natural products, holds promise as a key strategy for unlocking its higher nutritional and functional value.

This study aims to utilize two LAB strains isolated from yak yogurt—*L. bulgaricus* and *S. thermophilus*—to ferment PA powder and obtain the fermentation supernatant. To systematically evaluate its potential for alleviating UC, we adopted a four-pronged integrated research strategy involving: (i) Assessing the safety (hemolytic activity, antibiotic resistance) and functionality (antimicrobial activity) of the strains to ensure their suitability as functional starter cultures; (ii) Employing liquid chromatography–mass spectrometry (LC-MS)-based untargeted metabolomics to comprehensively characterize differences in metabolites before and after fermentation; (iii) Systematically evaluating the in vivo efficacy of the fermentation supernatant in intervening UC using a dextran sulfate sodium (DSS)-induced colitis mouse model, along with its anti-colon cancer efficacy through in vitro cell assays; (iv) Conducting whole-genome sequencing and analysis of the employed strains to comprehensively elucidate their genomic features, key metabolic pathways, and the genetic basis underlying health benefits, thereby providing molecular evidence for strains’ safety and functionality. This study not only contributes to exploring high-value transformation pathways for insect resources but also provides a theoretical basis and potential directions for application in developing novel anti-inflammatory functional products based on LAB fermentation strategies.

## 2. Materials and Methods

### 2.1. Experimental Strains

*L. bulgaricus* SN22 and *S. thermophilus* SN05 were isolated from yak yogurt. The strains were preserved by the China General Microbiological Culture Collection Center (CGMCC) under conditions of liquid nitrogen, −80 °C freezing, and vacuum freeze-drying. The seven foodborne pathogenic bacteria used in the antibacterial assays—*Escherichia coli* CMCC(B) 44102, *Salmonella Paratyphi B* CMCC 50094, *Pseudomonas aeruginosa* CMCC(B) 10104, *Salmonella Typhimurium* ATCC 14028, *Shigella dysenteriae* ATCC 51334, *Yersinia enterocolitica* ATCC 23715, and *Staphylococcus aureus* CMCC(B) 50094—along with the control strain *Lactobacillus rhamnosus* GG ATCC 53103 (LGG), were purchased from HuanKai Biotechnology (Shaoguan, China).

### 2.2. In Vitro Probiotic Evaluation of the Strains

The hemolysis assay was performed on Columbia blood agar plates (Kailing Trading Co., Ltd., Jiangmen, China). After incubation at 37 °C for 24 h, the presence of a hemolytic zone around the colonies was observed. Antimicrobial susceptibility testing was performed using the Kirby-Bauer disk diffusion method [27]. Seven antimicrobial disks (tetracycline, ampicillin, ceftriaxone, clarithromycin, gentamicin, chloramphenicol, and clindamycin) were purchased from Yuanye Bio-Technology Co., Ltd. (Shanghai, China). The bacterial suspension was adjusted to a concentration of 1 × 10^8^ CFU/mL and uniformly inoculated onto MRS agar plates using a sterile cotton swab. After air-drying at room temperature for 10 min, antimicrobial disks were aseptically placed on the plates. Following incubation at 37 °C for 24 h, the diameters of the inhibition zones were measured using a vernier caliper (Meinaite Industrial Co., Ltd., Shanghai, China). All experiments were performed in triplicate. The results were interpreted according to the CLSI (Clinical and Laboratory Standards Institute) guidelines, and the susceptibility of the strains was categorized as susceptible (S), intermediate (I), or resistant (R) [28].

The antimicrobial activity of the strains was assessed using the Oxford cup method [29] After cooling the sterilized nutrient agar to 40 °C, the target pathogenic bacterial suspension was thoroughly mixed and poured into plates. Following solidification, sterile Oxford cups were placed on the agar surface, and 50 μL of the test bacterial suspension (1 × 10^8^ CFU/mL) was added to each cup, with LGG serving as the control. The plates were incubated at 37 °C for 24 h, after which the diameters of the inhibition zones around the Oxford cups were measured using a vernier caliper. Three replicates were performed for each experimental group.

### 2.3. Preparation of Periplaneta americana Powder Fermentation Broth

PA powder was provided by Good Doctor Pharmaceutical Group Co., Ltd. (Chengdu, China; Pharmaceutical Production License No.: Chuan 20160179). The powder was prepared as follows: PA was dissolved in hot water at 55 °C–65 °C, followed by rinsing, draining, and immediate drying. Impurities were removed, and the material was further dried and ground into powder. Its main chemical characteristics include proteins and amino acids, nucleosides, lipids and fatty acids, carbohydrates, polypeptides, and other components.

SN22 and SN05 strains were individually inoculated into a medium containing 10 g PA powder, 2 g fructooligosaccharides, and 100 mL sterilized water, followed by static fermentation at 37 °C for 96 h. After fermentation, the samples were centrifuged at 4000 rpm and 4 °C for 10 min, and the supernatant was filtered through a 0.22 μm filter. The experimental groups (SN22-PA supernatant, SN05-PA supernatant) and the unfermented control group (PA) were set up with three replicates each.

### 2.4. Non-Targeted Metabolomics Profiling

Processed samples (refer to Section 2.3) were subjected to non-targeted metabolomic analysis performed by Novogene Bioinformatics Technology Co., Ltd. (Beijing, China). The sample (1 mL) was lyophilized using a lyophilizer (Licheng Instrument Technology Co., Ltd., Changsha, China). The lyophilized material was then resuspended in 100 μL of 80% (*v*/*v*) methanol aqueous solution, followed by vortexing for 30 s and subsequent incubation on ice for 5 min. The mixture was centrifuged at 15,000× *g* for 15 min at 4 °C. An appropriate volume of the supernatant was diluted with mass spectrometry-grade water to achieve a final methanol concentration of 53% (*v*/*v*). The diluted solution was recentrifuged under identical conditions (15,000× *g*, 15 min, 4 °C). The resulting supernatant was collected and subjected to LC-MS analysis. The mass spectrometer was operated with a scan range of m/z 100–1500. Electrospray ionization (ESI) source parameters were configured as follows: spray voltage, 3.5 kV; sheath gas flow rate, 35 psi; auxiliary gas flow rate, 10 L/min; capillary temperature, 320 °C; S-lens RF level, 60; auxiliary gas heater temperature, 350 °C. Analyses were performed in both positive and negative ion modes. MS/MS fragmentation was conducted using data-dependent scans for targeted precursor ions.

### 2.5. Animals and Treatments

Thirty 4-week-old male Kunming mice (specific pathogen-free [SPF] grade, body weight approximately 20 ± 2 g) were purchased from Dashuo Experimental Animal Co., Ltd. (Chengdu, China) (Production License No. SCXK [Chuan] 2020-0030). The mice were randomly allocated into 10 cages with 3 animals per cage. Mice were housed under sterile conditions with a temperature maintained at 20 ± 2 °C, humidity fixed at 45 ± 10%, and a 12 h light/dark cycle. After one week of acclimatization, mice were randomly divided into five groups (*n* = 6 in each group): the Blank Control group (Control) received daily gavage with 0.2 mL normal saline for 14 days; the Model group (DSS Control) received daily gavage with 0.2 mL normal saline for 14 days; the Unfermented Control group received daily gavage with 0.2 mL PA supernatant; the Prevention group SN05 + PA received daily gavage with 0.2 mL SN05-PA supernatant for 14 days; the Prevention group SN22 + PA received daily gavage with 0.2 mL SN22-PA supernatant for 14 days. Except for the Control group, which drank sterile water freely, the other four groups drank 4% DSS solution freely starting from day 7.

During the experimental period, the activity status, fecal condition, and presence of hematochezia were observed daily. The Disease Activity Index (DAI) was scored according to the criteria established by Manicassamy and Manoharan [30]. At the end of the experiment, three mice from each group were randomly selected for euthanasia, and orbital blood was collected for the detection of serum inflammatory cytokines. Fresh blood samples were centrifuged at 3000 rpm for 10 min to obtain serum. The levels of serum inflammatory cytokines (IL-1β, IL-6, TNF-α, and IL-10) were measured using ELISA kits (Jingmei Biotechnology Co., Ltd., Yancheng, China). The procedures were performed according to the manufacturer’s instructions. After orbital blood collection, the colorectum was immediately dissected and measured, then immersed in 4% paraformaldehyde fixative, and sent to Lilai Biotechnology Co., Ltd. (Chengdu, China) for H&E staining and histopathological examination. All experimental procedures were conducted in accordance with the ethical guidelines for animal experimentation of Sichuan University (SCU251024001).

### 2.6. Cytotoxicity Assay on HT-29 Cells

#### 2.6.1. Determination of Growth Inhibitory Effect of Fermented Supernatant on HT-29 Cells

Human colon cancer HT-29 cells were seeded into 96-well plates at a density of 3 × 10^4^ cells per well and cultured to allow adherence. After adherence, the medium was discarded. The experimental groups received 100 μL of serum-free medium containing 4% (*v*/*v*) fermented supernatant (prepared as described in Section 2.3), while the control groups received an equal volume of serum-free medium alone. Each group was set up in triplicate and incubated for 24, 48, and 72 h, respectively. Following incubation, the culture medium was removed, and 100 μL of CCK-8 solution was added to each well. The plates were then incubated for an additional 90 min. Finally, the optical density (OD) at 562 nm was measured using a microplate reader (Thermo Fisher Scientific, Inc., Waltham, MA, USA) to assess cell viability and determine the growth inhibitory effect of the fermented supernatant.

#### 2.6.2. Effect of Fermented Supernatant on HT-29 Cells Colony Formation Ability

The HT-29 cells were seeded into 6-well plates at a density of 2000 cells per well. For 3 weeks, the cells were cultured in serum-free medium containing 1% (*v*/*v*) fermented supernatant. The medium was replaced with fresh medium containing the same concentration of supernatant every two days to maintain cell condition. After the cultivation period, the medium was discarded and the cells were gently washed with phosphate-buffered saline (PBS). The cells were then fixed with 4% paraformaldehyde (PFA) for 15–20 min at room temperature, followed by staining with 1% crystal violet solution for 30 min. After staining, the plates were thoroughly rinsed with distilled water to remove residual stain and air-dried. Macroscopically visible cell colonies were counted under a microscope. The experiment was performed in triplicate.

#### 2.6.3. Effect of Fermented Supernatant on Reactive Oxygen Species (ROS) Levels in HT-29 Cells

HT-29 cells adherent cells were treated with fermented supernatant for 48 h. Subsequently, the cells were gently detached using a pipette (Thermo Fisher Scientific, Inc., Waltham, MA, USA), harvested, and washed twice with serum-free medium by centrifugation to obtain a cell pellet. The cell pellet was resuspended in probe solution containing 10 μM 2′,7′-dichlorodihydrofluorescein diacetate (DCFH-DA) (diluted 1:1000 in serum-free medium) and adjusted to a concentration of approximately 1 × 10^7^ cells/mL. The cell suspension was then incubated at 37 °C for 20 min in the dark. Following incubation, the cells were washed three times with serum-free medium to thoroughly remove the extracellular DCFH-DA probe. Finally, cellular fluorescence intensity was visualized and measured using an inverted fluorescence microscope (Nikon Corporation, Tokyo, Japan) at excitation/emission (Ex/Em) wavelengths of 488 nm and 525 nm, respectively. Cells treated with non-fermented supernatant served as the control group. The experiment was performed in triplicate.

#### 2.6.4. Detection of Apoptosis by TUNEL Assay

After treatment with fermented supernatant for 48 h, adherent HT-29 cells were subjected to TUNEL assay. Briefly, the culture supernatant was discarded and the cells were washed with PBS. The cells were then fixed with 4% PFA for 30 min at room temperature, followed by another PBS wash. Subsequently, the cells were permeabilized with PBS containing 0.3% Triton X-100 (Beyotime Biotechnology Co., Ltd., Shanghai, China) for 5 min at room temperature. Following permeabilization, the cells were incubated with 50 μL of TUNEL reaction mixture at 37 °C in the dark for 60 min. After incubation, the cells were washed three times with PBS. Finally, the cells were mounted with anti-fade mounting medium and visualized under an inverted fluorescence microscope. Apoptotic cells exhibiting green fluorescence were detected at excitation/emission (Ex/Em) wavelengths of 550 nm and 570 nm, respectively. The experiment was performed in triplicate.

### 2.7. Genome Analysis

#### 2.7.1. DNA Extraction and Whole Genome Sequencing

The bacterial genomic DNA of SN22 and SN05 was extracted using a Bacterial Genomic DNA Extraction Kit (Tiangen Biotechnology Co., Ltd., Beijing, China). The quality of the extracted genomic DNA was subsequently assessed with a NanoDrop 2000 UV-Vis spectrophotometer (Thermo Fisher Scientific, Inc., Waltham, MA, USA) and a Qubit 2.0 fluorometer (Life Technologies, Carlsbad, CA, USA). Upon confirmation of qualified DNA integrity, SMRTbell libraries were constructed. Sequencing was performed by Berry Genomics Co., Ltd., (Beijing, China) using the PacBio Sequel II/IIe/Revio platform.

To obtain complete genome sequences, high-quality HiFi reads were subjected to de novo assembly using the Hifiasm software (v0.19.5). Given the typical circular architecture of bacterial genomes, overlapping sequences between the start and end positions were trimmed to generate closed circular genomes. The accuracy of the assembled genomes was validated by aligning HiFi reads to the draft genomes through the pbmm2 software (v1.16.0), which provided coverage information [31]. Genome assembly completeness and integrity were further evaluated by analyzing conserved genes in the BUSCO database [32].

#### 2.7.2. Phylogenetic Tree Construction and Average Nucleotide Identity (ANI) Analysis

Genomic assemblies of 14 *L. bulgaricus* and 14 *S. thermophilus* strains were retrieved from the NCBI public database. Orthologous genes were annotated and extracted using OrthoFinder (v3.0.1b1). All single-copy orthologous genes were subjected to multiple sequence alignment with MAFFT (v7.505), followed by trimming of low-quality regions using TrimAI (v1.4.rev15). A maximum-likelihood phylogenetic tree was constructed with IQ-TREE (v2.4.0) and visualized on the iTOL online platform. For pairwise genomic comparisons, the Average Nucleotide Identity (ANI) values between any two strains were calculated using fastANI (v1.34). The resulting ANI matrix was used to generate a heatmap for comparative genomic analysis.

#### 2.7.3. Gene Prediction and Functional Annotation

Coding genes in the bacterial genomes were predicted using Prodigal. tRNA genes were identified with tRNAscan-SE, while rRNA loci were localized by aligning homologous rRNA sequences to the assembled genomes via BLAST (v2.2.25). Non-coding RNA (ncRNA) annotations were further refined by aligning sequences to the Rfam database using BLAST. The obtained gene sequences were subjected to BLAST analysis against universal functional databases—including the Kyoto Encyclopedia of Genes and Genomes (KEGG), Gene Ontology (GO), and Clusters of Orthologous Groups (COG)—using Prodigal software (v2.6.3) to acquire functional annotation results. Antibiotic resistance genes were annotated by comparing protein sequences to the Comprehensive Antibiotic Resistance Database (CARD) through BLAST analysis. Carbohydrate-active enzymes were identified using dbCAN3 with the CAZy database as a reference. Secondary metabolite biosynthesis gene clusters, including potential bacteriocin synteny, were predicted using antiSMASH (v8.0). Finally, a circular genome map was generated with CGview, integrating coding genes (strand-specific), COG functional categories, rRNA/tRNA elements, GC skew dynamics, and GC content distribution to visualize structural features of the genome.

### 2.8. Statistical Analysis

All experiments were performed in triplicate. Data are presented as mean ± standard deviation (SD). Statistical analyses were conducted using GraphPad Prism 8 (GraphPad Software, Inc., San Diego, CA, USA). Experimental results were compared using one-way analysis of variance (ANOVA) followed by post hoc tests, with significance set at *p* < 0.05.

After preprocessing with the metaX software (v1.4.17), the metabolomics data were subjected to principal component analysis (PCA) and partial least squares discriminant analysis (PLS-DA) to obtain the variable importance in projection (VIP) values for each metabolite. PCA, volcano plots, clustering heatmaps, and correlation network diagrams were all generated using R software (version 4.4.2). Significantly different metabolites between groups were identified using the following criteria: VIP > 1.0, fold change (FC) > 1.5 or FC < 0.667, and *p* < 0.05. The identified differential metabolites were annotated and their pathways were analyzed using the KEGG.

## 3. Results

### 3.1. Safety Assessment of SN22 and SN05 and Their Inhibitory Effects Against Foodborne Pathogens

**Figure 1:**
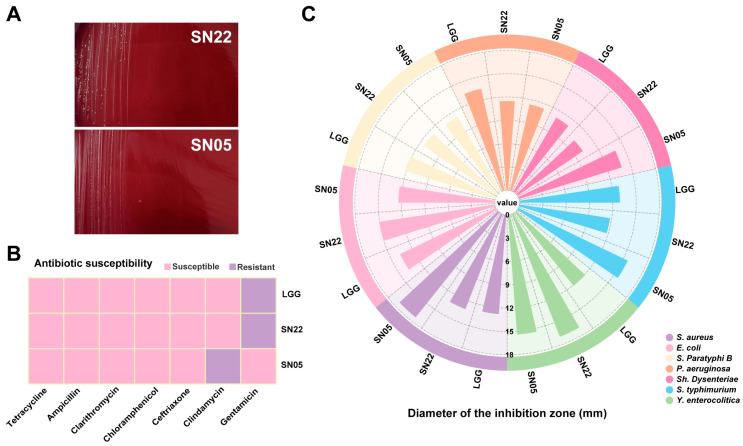
In vitro probiotic evaluation of SN22 and SN05. (**A**) Hemolysis assessment of strains SN22 and SN05. (**B**) Antibiotic susceptibility testing of strains LGG, SN22, and SN05 against common antibiotics. (**C**) Antimicrobial activity assessment of strains LGG, SN22, and SN05.

Hemolysis assays revealed no hemolytic activity for either strain SN22 or SN05 (Figure 1A). In the antibiotic susceptibility testing, both strains showed susceptibility to a variety of common antibiotics (such as tetracycline, ampicillin, ceftriaxone, clarithromycin, and chloramphenicol), indicating broad-spectrum antibiotic sensitivity. However, it is noteworthy that strain SN22 was resistant to gentamicin, while SN05 was resistant to clindamycin (Figure 1B). In vitro antimicrobial assays systematically evaluated the inhibitory spectrum against seven foodborne pathogens. Both strains exhibited significant growth inhibition against Gram-negative and Gram-positive bacteria, with inhibition zone diameters comparable to the reference strain LGG. Notably, SN22 showed stronger inhibition against *Y. enterocolitica* (16.88 ± 0.09 mm vs. LGG 12.82 ± 0.72 mm, *p* < 0.01), and SN05 demonstrated superior efficacy against *S. aureus* (18.30 ± 0.89 mm vs. LGG 13.09 ± 0.49 mm, *p* < 0.01) (Figure 1C). Based on the above results, SN22 and SN05 demonstrated favorable characteristics in both basic safety (hemolytic activity) and antimicrobial activity. However, the gentamicin and clindamycin resistance they carry requires further evaluation in subsequent studies to assess the risk of transferability. Given their notable antimicrobial potential, they represent promising candidate strains for the fermentation of PA under controlled conditions and warrant further in-depth investigation.

### 3.2. Fermentation Significantly Reshapes the Metabolome Profile of Periplaneta americana Powder

**Figure 2:**
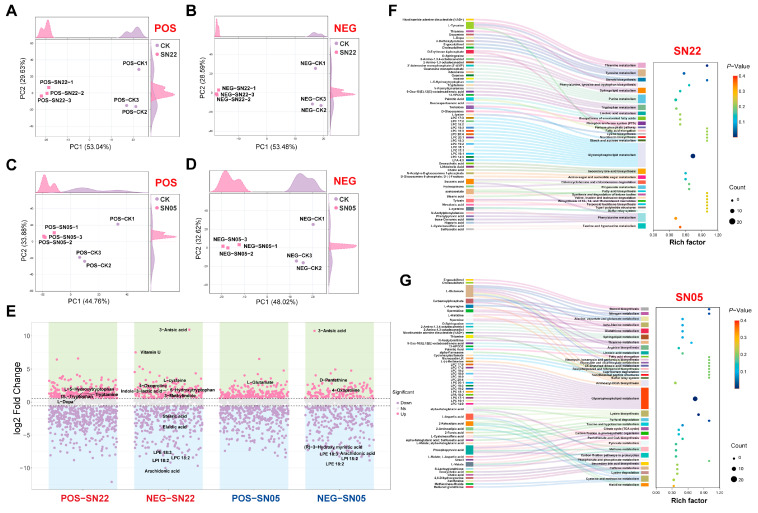
Metabolic analysis and pathway enrichment analysis of *Periplaneta americana* powder fermented by SN22 and SN05 in positive and negative ion modes. (**A**–**D**) PCA of fermented groups vs. control for SN22 and SN05 in positive and negative ion modes. (**E**) Differential metabolite screening between fermented groups (SN22, SN05) and the control in positive and negative ion modes. (**F**,**G**) KEGG pathway enrichment analysis of *Periplaneta americana* powder fermented by SN22 and SN05.

PCA analysis confirmed that fermentation of PA powder with SN22 and SN05, respectively, resulted in distinct metabolite compositions compared to the control group. Clear separation was observed between both fermented groups and the control group along the principal components in both positive and negative ion modes. For the SN22 group, PC1 and PC2 collectively explained 82.67% (positive mode) and 82.07% (negative mode) of the total variance. Similarly, for the SN05 group, PC1 and PC2 together accounted for 78.64% (positive mode) and 80.64% (negative mode) of the total variance, demonstrating that the fermentation process significantly altered the metabolite features of PA powder (Figure 2A–D).

Metabolomic analysis revealed that fermentation of PA powder by strains SN22 and SN05 significantly altered the metabolite profiles. After 96 h of fermentation with SN22, 463 differential metabolites were identified compared to the control group (172 upregulated, 291 downregulated), primarily distributed within the categories of lipids and lipid-like molecules (26.57%), organic acids and derivatives (11.45%), and organoheterocyclic compounds (6.48%) (Figure 2E). Specifically, glycerophospholipid components (e.g., lyso-phosphatidylinositol LPI 18:2, VIP = 1.62, fold change (FC) = 0.0029; lyso-phosphatidylcholine LPC 18:2, VIP = 1.47, FC = 0.0034; lyso-phosphatidylethanolamine LPE 18:3, VIP = 1.63, FC = 0.0037; *p* < 0.05) and fatty acid metabolites (e.g., arachidonic acid VIP = 1.60, FC = 0.0010; elaidic acid VIP = 1.33, FC = 0.0541; stearic acid VIP = 1.44, FC = 0.1661; *p* < 0.05) exhibited systematic downregulation; while amino acid substances (e.g., Vitamin U VIP = 1.11, FC = 180.89; L-cysteine VIP = 1.40, FC = 7.26; 4-oxoproline VIP = 1.39, FC = 4.41; *p* < 0.05) and neurotransmitter precursors (L-dopa VIP = 1.40, FC = 2.88; *p* < 0.05) accumulated significantly. Products related to the tryptophan metabolism pathway (e.g., 5-hydroxytryptophan VIP = 1.48, FC = 4.07; indole-3-lactic acid VIP = 1.28, FC = 3.67; tryptamine VIP = 1.20, FC = 2.45; *p* < 0.05) were significantly upregulated. Similarly, fermentation with SN05 resulted in 292 differential metabolites (99 upregulated, 193 downregulated), predominantly regulated within the categories of fatty acids (31.5%), amino acids (25.7%), and terpenoids (12.0%) (Figure 2E). Notably, both fermented groups showed a significant decrease in arachidonic acid content (SN22: FC = 0.0010; SN05: FC = 0.0031), overexpression of 3-anisic acid (SN22: FC = 1960.41; SN05: FC = 1731.14), and accumulation of 4-oxoproline (SN22: FC = 4.41; SN05: FC = 4.74).

KEGG enrichment analysis of differential metabolites after separate fermentation with SN22 and SN05 revealed that glycerophospholipid metabolism was significantly enriched in both groups. Significant alterations were observed for multiple phospholipid compounds within this pathway, such as LPC and LPA, in both fermented samples (Figure 2F,G). Furthermore, pathways significantly enriched specifically in the SN22 group included thiamine metabolism, tyrosine metabolism, steroid biosynthesis, secondary bile acid biosynthesis, and amino sugar and nucleotide sugar metabolism (Figure 2F). In contrast, the SN05 group showed significant enrichment in steroid biosynthesis, nitrogen metabolism, and alanine, aspartate, and glutamate metabolism (Figure 2G).

### 3.3. The Ameliorative Effect of Fermentation Supernatant on DSS-Induced Colitis in Mice

**Figure 3:**
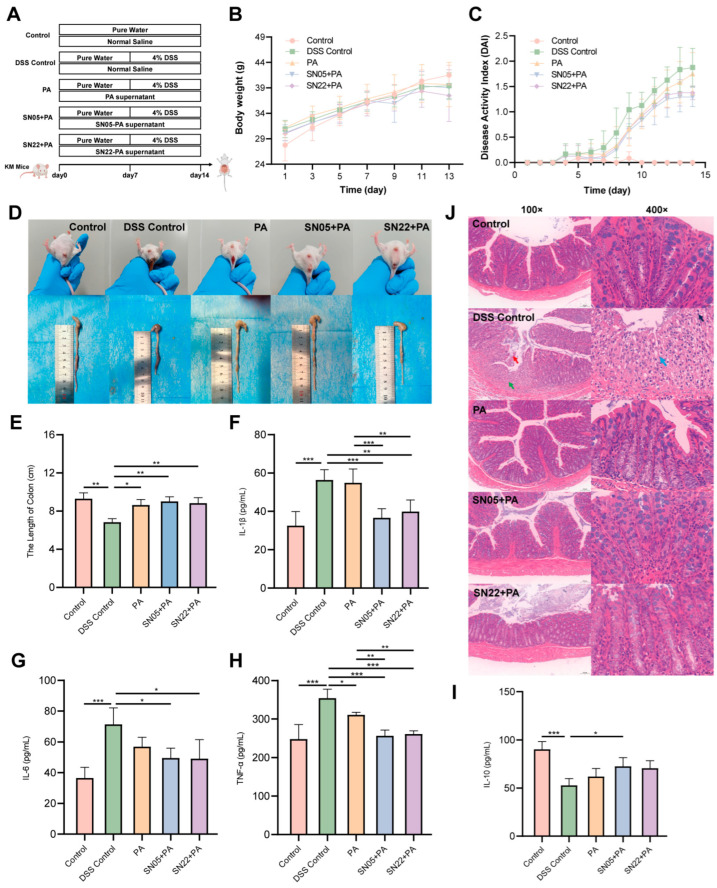
Overall therapeutic effects of fermented supernatant on DSS-induced UC mice and intestinal pathology. (**A**) Schematic diagram of the animal experimental design. (**B**) Body weight changes in each group. (**C**) DAI scores. (**D**) Rectal bleeding status and representative images of colon tissues on day 14. (**E**) Colon lengths of mice in each group. (**F**–**I**) Serum levels of inflammatory cytokines: IL-1β (**F**), IL-6 (**G**), TNF-α (**H**), and IL-10 (**I**). (*: *p* < 0.05, **: *p* < 0.01, ***: *p* < 0.001). (**J**) H&E staining of colon tissues in each group. Scale bar: 100 μm. Red arrow: shedding of mucosal epithelial cells. Green arrow: degeneration and necrosis of the lamina propria. Blue arrow: fibroblasts. Black arrow: neutrophils.

To further investigate the therapeutic efficacy of PA powder fermentation supernatants on UC mice, a murine UC model was established using 4% DSS, followed by intervention with the prepared samples (Figure 3A). Body weight monitoring showed weight loss starting from day 11 in all groups compared to the Control group (Figure 3B). DAI scores revealed a continuous increase over time in the DSS Control group. From day 8 onward, both fermented groups exhibited lower DAI scores than the unfermented PA group (Figure 3C). Experimental observations revealed that mice in the DSS Control and unfermented PA groups commonly displayed symptoms including lethargy, reduced activity, diarrhea, and hematochezia, whereas symptoms in both fermented groups were markedly milder, with less frequent hematochezia; furthermore, the colon lengths in both fermented groups were comparable to the Control group (Figure 3D). Measurement of colon length confirmed that treatment with both fermented groups resulted in colon lengths similar to the Control group, unlike the significantly shortened colons in the DSS Control group (Figure 3E). Serum ELISA results indicated that compared to the DSS Control group, both fermented groups significantly reduced levels of the pro-inflammatory cytokines IL-1β, IL-6, and TNF-α (*p* < 0.05), with the SN05 + PA group also showing a significant increase in the anti-inflammatory cytokine IL-10 (*p* < 0.05). Furthermore, both fermented groups exhibited significantly lower serum levels of IL-1β and TNF-α compared to the unfermented group (*p* < 0.05) (Figure 3F–I). HE staining revealed mucosal epithelial cell sloughing, degeneration, and necrosis in the lamina propria, and marked fibroplasia in the DSS Control group. In contrast, both fermented treatment groups maintained largely intact colonic architecture: the mucosal epithelium was orderly arranged without significant sloughing; crypt structures were clear with abundant goblet cells; and the lamina propria showed only minimal scattered inflammatory cell infiltration without significant pathological alterations, indicating that intervention with the fermented supernatants effectively alleviated DSS-induced colonic mucosal damage (Figure 3J). Immunohistochemical results (Table 1) showed that compared to the DSS Control group, both fermented groups exhibited significantly downregulated positive expression of the pro-inflammatory cytokines IL-1β, IL-6, and TNF-α (*p* < 0.05) in colon tissue, alongside significantly upregulated expression of the anti-inflammatory cytokine IL-10 (*p* < 0.05). Compared to the unfermented PA group, both fermented groups demonstrated further reduced expression of IL-1β, IL-6, and TNF-α and increased expression of IL-10, with SN05 showing significant reductions in IL-6 and TNF-α (*p* < 0.05), suggesting that fermentation significantly suppressed local colonic pro-inflammatory mediator expression and enhanced the production of the anti-inflammatory factor IL-10, consistent with the serum ELISA results.

### 3.4. Growth Inhibitory and Pro-Apoptotic Effects of Fermentation Supernatants on HT-29 Cells

**Figure 4:**
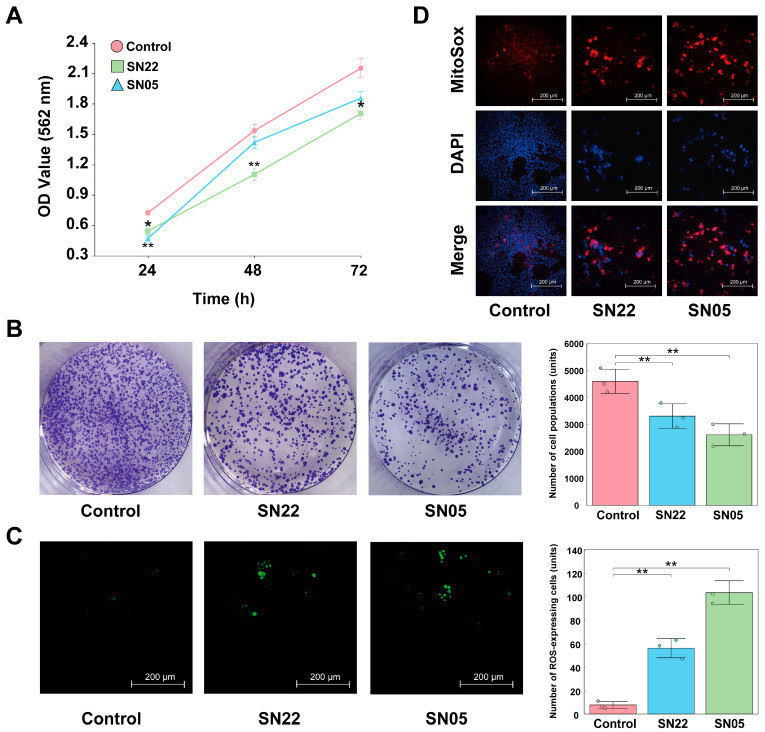
Exploring the mechanism of HT-29 cell inhibition by SN22/SN05 fermentation supernatant. (**A**) Growth inhibitory effects of fermentation supernatant on HT-29 cells (*: *p* < 0.05, **: *p* < 0.01). (**B**) Effects of fermentation supernatant on the colony formation ability of HT-29 cells (**: *p* < 0.01). (**C**) Effects of fermentation supernatant on ROS levels in HT-29 cells (**: *p* < 0.01). (**D**) Pro-apoptotic effects of fermentation supernatant on HT-29 cells assessed by TUNEL assay.

Cell experimental results demonstrated that both fermented supernatants (SN22 and SN05) significantly inhibited the growth of HT-29 cells (*p* < 0.05). While cells in the Control group proliferated continuously over 72 h, those treated with SN22 and SN05 exhibited reduced growth compared to the Control starting at 24 h (Figure 4A). Colony formation assays revealed that treatment with SN22 and SN05 fermented supernatants significantly impaired the colony-forming ability of HT-29 cells relative to the Control (*p* < 0.05), reducing the average number of colonies by 28.07% and 43.14%, respectively (Figure 4B). Furthermore, fermented supernatant treatment significantly elevated intracellular ROS levels in HT-29 cells (*p* < 0.05), with SN22 and SN05 groups showing increases of 7.30-fold and 13.48-fold compared to the Control group (Figure 4C). TUNEL assay results indicated that the fermented supernatants significantly induced HT-29 cell apoptosis. Fluorescence intensity, indicative of apoptosis, was markedly higher in the SN22 and SN05 groups, showing increases of 9.27-fold and 13.53-fold compared to the Control group (Figure 4D).

### 3.5. Genomic Features of the Two Strains

Genomic characteristics statistics (Table 2) revealed that *L. bulgaricus* SN22 and *S. thermophilus* SN05 generated 152,407 and 72,574 sequencing reads, totaling 1,198,293,806 and 495,867,121 base pairs (bp), respectively. The average read lengths were 7862.46 bp and 6832.57 bp, with N50 values of 8820 and 7669, indicating high-quality datasets suitable for genome assembly. HiFi reads were assembled using Hifiasm, yielding single-contig genomes for both strains. The maximum contig lengths, N50 values, and total contig lengths of the assembled genomes were 1,775,614 bp (SN22) and 1,945,731 bp (SN05), confirming their circular chromosomal architecture. The genomic GC content was 50.05% (SN22) and 39.06% (SN05), reflecting distinct genomic GC composition. Further analysis identified 1786 and 2034 protein-coding genes in SN22 and SN05, respectively, with average gene lengths of 835.29 bp and 798.23 bp. The assemblies demonstrated high continuity and completeness, providing a robust foundation for subsequent genomic analyses and functional investigations.

### 3.6. Phylogenetic Analysis and ANI

**Figure 5:**
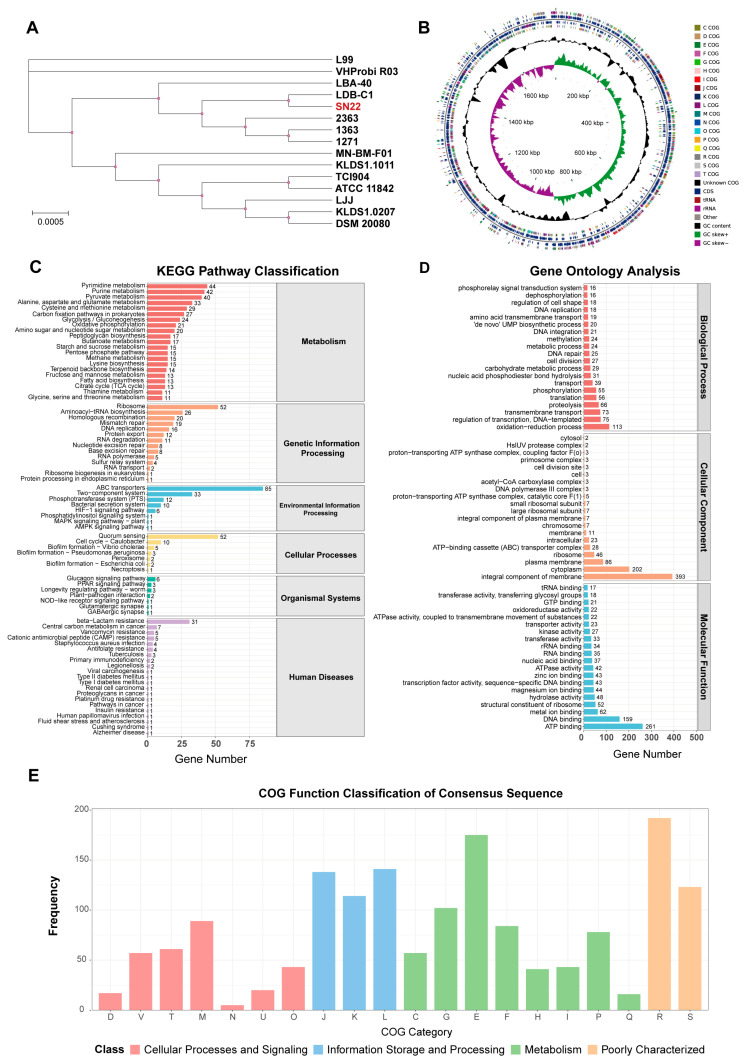
Phylogenetic tree and functional annotation of the *L. bulgaricus* SN22 genome. (**A**) Phylogenetic tree of SN22 and 14 conspecific strains based on core genome sequences. (**B**) Circular genomic map depicting gene locations and annotation results for the SN22 genome. (**C**–**E**) Functional annotation of the SN22 genome using NCBI Blast+: KEGG pathways (**C**), COG categories (**D**), and GO terms (**E**).

The ANI values between *L. bulgaricus* SN22 and 14 publicly available strains of the same subspecies, as well as between *S. thermophilus* SN05 and 14 reference strains, were calculated. All pairwise ANI values exceeded 98% (Figure S1A,B), confirming that SN22 and SN05 belong to their respective species with high intraspecies homogeneity. Most strains in the comparison were isolated from fermented dairy products. Phylogenetic trees were reconstructed using the genomes of SN22, SN05, and their conspecific strains (Figure 5A and Figure S2A). SN22 exhibited the closest phylogenetic relationship with *L. bulgaricus* LDB-C1 (ANI = 99.06%), which was isolated from fermented milk. Similarly, SN05 showed the highest ANI (98.88%) with *S. thermophilus* KLDS 3, a strain derived from fermented yogurt.

### 3.7. Gene Functional Annotation of the Two Strains

A total of 157 (8.79%) and 163 (8.01%) antibiotic resistance genes were annotated in *L. bulgaricus* SN22 and *S. thermophilus* SN05, respectively. However, alignment against the CARD revealed low average sequence similarities of 33.39 ± 8.16% and 33.63 ± 9.00% for these resistance-associated genes. Screening using the Best Hit Bitscore > Pass Bitscore criterion identified no validated resistance genes in SN22, while SN05 harbored vancomycin-related resistance genes: vanY (within the vanB cluster) and vanT (within the vanG cluster).

In the KEGG annotation, 1051 (58.85%) genes in SN22 and 1220 (59.98%) genes in SN05 were functionally assigned to primary metabolic pathways: Metabolism, Genetic Information Processing, Environmental Information Processing, Cellular Processes, Organismal Systems, and Human Diseases (Figure 5C and Figure S2C). Among these, genes associated with the metabolism pathway were the most abundant for both strains, accounting for 64.80% and 61.89% of their respective annotated genes, followed by genes involved in Genetic Information Processing, representing 17.60% and 15.08% of the annotated genes. Within the metabolism hierarchy, SN22 possessed 44 genes related to pyrimidine metabolism, 42 genes related to purine metabolism, and 40 genes related to pyruvate metabolism, while SN05 possessed 43 genes related to purine metabolism, 33 genes related to pyrimidine metabolism, and 32 genes related to glycolysis/gluconeogenesis. In the genetic information processing category, SN22 contained 52 genes associated with the ribosome pathway and 26 genes associated with aminoacyl-tRNA biosynthesis; SN05 contained 53 genes associated with the ribosome pathway and 27 genes associated with aminoacyl-tRNA biosynthesis.

The GO annotation results for SN22 and SN05 are shown in Figure 5D and Figure S2D. SN22 had 1474 genes (82.53%) assigned to GO functional categories, with 1098 genes in Biological Process (BP), 758 genes in Cellular Component (CC), and 1210 genes in Molecular Function (MF). Similarly, SN05 had 1492 genes (73.35%) assigned to GO categories, comprising 1086 BP genes, 782 CC genes, and 1236 MF genes (with some genes annotated in multiple categories). Within the BP category, “oxidation-reduction process” was the most prevalent term for both strains, accounting for 7.67% and 6.37% of their respective annotated genes in this category. For the CC category, “integral component of membrane” contained the highest number of genes in both strains, representing 26.66% (SN22) and 25.47% (SN05) of their respective CC-annotated genes. In the MF category, “ATP binding” was the term with the most assigned genes for both strains, comprising 17.70% (SN22) and 16.29% (SN05) of their respective MF-annotated genes.

Comparison against the COG database predicted 1438 (80.52%) and 1757 (86.38%) functionally annotated genes for strains SN22 and SN05, respectively. Within the metabolism category, both strains exhibited the highest number of genes associated with “Amino acid transport and metabolism” (E), accounting for 175 genes (12.17% of its COG genes) in SN22 and 211 genes (12.01% of its COG genes) in SN05. This was followed by genes related to “Carbohydrate transport and metabolism” (G), with 102 genes (7.09%) in SN22 and 112 genes (6.37%) in SN05 (Figure 5E and Figure S2E).

### 3.8. Genomic Features of Secondary Metabolites and CAZymes

**Figure 6:**
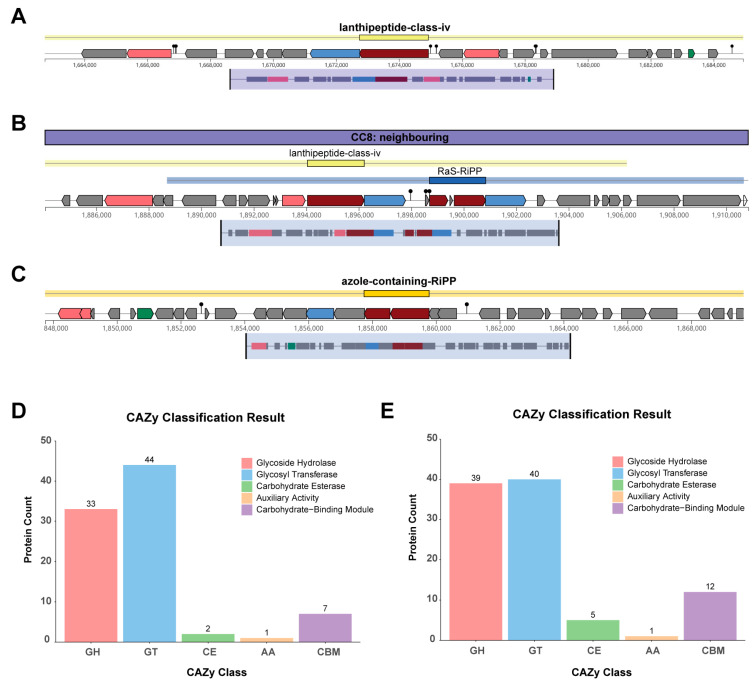
Metabolic system research on SN22 and SN05. (**A**) Secondary metabolite biosynthetic gene clusters (BGCs) predicted by antiSMASH in the SN22 genome. (**B**,**C**) Secondary metabolite biosynthetic gene clusters (BGCs) predicted by antiSMASH in the SN05 genome. Color legend: red indicates core biosynthetic genes, pink indicates additional biosynthetic genes, blue indicates transport-related genes, green indicates regulatory genes, and gray indicates other genes. (**D**,**E**) CAZy annotation of the SN22 (**D**) and SN05 (**E**) genomes using dbCAN.

Genome mining identified a Lanthipeptide-class-IV bacteriocin biosynthetic gene cluster in both strains SN22 and SN05 (Figure 6A,B). Additionally, an Azole-containing-RiPP bacteriocin biosynthetic gene cluster was predicted in SN05 (Figure 6C). Annotation against the CAZymes database revealed that both strains possess genes encoding key functional modules, including Glycoside Hydrolases (GHs), Glycosyl Transferases (GTs), Carbohydrate Esterases (CEs), Auxiliary Activities (AAs), and Carbohydrate-Binding Modules (CBMs). Among these, GTs were the most abundant category in both strains, accounting for 50.57% and 41.24% of the total annotated CAZymes genes in SN22 and SN05, respectively. This was followed by GHs, representing 37.93% (SN22) and 40.21% (SN05) of the annotated CAZymes genes (Figure 6D,E).

## 4. Discussion

The safety assessment of strains is an essential prerequisite for their application in the fields of food and pharmaceuticals [33,34]. Hemolytic activity is one of the critical indicators for evaluating microbial safety [35]. In this study, both *L. bulgaricus* SN22 and *S. thermophilus* SN05 exhibited no hemolytic activity, which is consistent with the safety requirements for microbial strains [36]. Additionally, with the growing problem of antibiotic resistance, selecting strains that are sensitive to antibiotics is also crucial to prevent the spread of resistance genes [37]. In this study, both strains demonstrated broad antibiotic susceptibility. However, SN22 exhibited phenotypic resistance to gentamicin, while SN05 showed resistance to clindamycin. Since phenotypic resistance testing alone cannot fully evaluate the risk of antibiotic resistance spread, we performed a comprehensive whole-genome analysis to assess their safety. Whole-genome sequencing and antibiotic resistance genes (ARGs) analysis indicated that the observed resistance was intrinsic. Resistance to gentamicin in *L. bulgaricus* and to clindamycin in *S. thermophilus* is commonly found in these species and is generally considered intrinsic [38]. This type of resistance originates from specific bacterial membrane structures and the absence of cytochrome-mediated electron transport systems required for antibiotic uptake [39,40]. This type of resistance poses a minimal risk of transmission. Comparative analysis against databases such as CARD revealed that the predicted ARGs located on the chromosome showed generally less than 80% amino acid sequence identity to known functional resistance genes, falling below the commonly used threshold for homology-based functional assignment [41]. These findings suggest that the predicted genes are more likely to be non-functional pseudogenes or homologous sequences rather than functional resistance genes. Furthermore, no plasmids were detected in either strain, indicating a lack of mobile genetic elements that could facilitate the horizontal transfer of antibiotic resistance genes. Therefore, despite the observed phenotypic resistance, the risk of dissemination is considered very low. In summary, the risks associated with using these two strains for the production of bioactive substances under controlled non-clinical conditions (such as in the fermentation process described in this study) are considered manageable.

Genomic analysis revealed that the Lanthipeptide-class-IV gene cluster shared by the two strains belongs to a typical ribosomally synthesized and post-translationally modified peptide (RiPPs). Its products are characterized by lanthionine rings formed through thioether bridges. These compounds can selectively inhibit competing microorganisms, thereby providing the host with an advantage in ecological niche competition [42]. Through a review of the relevant literature and systematic comparison of multiple complete genomes of *L. bulgaricus* and *S. thermophilus* obtained from the NCBI database, it was found that the Lanthipeptide-class-IV bacteriocin gene cluster is widely present in both species [43,44]. On the other hand, the Azole-containing-RiPP gene cluster unique to strain SN05 indicates its specificity at the strain level. These compounds exert antimicrobial activity through multiple mechanisms, including interfering with cell wall synthesis, inhibiting protein translation, and disrupting membrane integrity [45]. The presence of these two bacteriocin gene clusters provides a potential molecular basis for the observed inhibition of *Y. enterocolitica* by strain SN22 and *S. aureus* by strain SN05 in antibacterial assays. This genomically encoded antibacterial capacity not only helps ensure sterility during fermentation and reduces the risk of microbial contamination, but also suggests that their metabolites may continue to exert antimicrobial effects in the gut microenvironment, thereby inhibiting pathogen colonization and promoting the restoration of intestinal microbiota homeostasis [46]. However, it must be noted that although this study proposed the above hypothesis through genomic comparison and phenotypic correlation, direct evidence from functional genetic validation is still lacking. Due to bottlenecks in current CRISPR gene editing technology in LAB, such as low transformation efficiency and difficulties in homologous recombination—particularly for multisite knockout of large gene clusters—this study could not verify the function of the cluster through gene deletion experiments. Thus, a causal relationship between genotype and phenotype could not be established, which represents a significant limitation of this study. Future research will prioritize the use of CRISPR-based genome editing technology to functionally validate the aforementioned bacteriocin gene clusters and clarify their specific products and antibacterial mechanisms. In addition, transcriptomic and metabolomic analyses will be applied to further elucidate their expression and regulatory networks during fermentation and under simulated gut conditions. This will provide higher-level evidence regarding the physiological relevance and application potential of these genetic traits.

Untargeted metabolomic analysis revealed that mono-strain fermentation by either *L. bulgaricus* SN22 or *S. thermophilus* SN05 significantly reshaped the metabolic profile of PAP supernatant. This metabolic remodeling represents a crucial step in the dairy-starter-mediated bioconversion of the raw insect matrix into high-value functional products. Differential metabolite analysis demonstrated a bidirectional modulation pattern during fermentation: Firstly, arachidonic acid (AA) content plummeted by 99.9%, drastically reducing the precursor pool for synthesizing potent pro-inflammatory mediators like prostaglandins and leukotrienes [47], thereby substantially diminishing the pro-inflammatory potential of the fermented supernatant. Conversely, bioactive components such as 3-anisic acid, vitamin U, L-dopa, and 5-hydroxytryptophan were significantly enriched, implicated in anti-inflammatory effects [48], antioxidant/mucosal repair (via Nrf2/Hmox1 pathway activation) [49], and potential neuro-immunomodulatory functions [50,51], respectively. It should be noted that directly linking the aforementioned specific differential metabolites (e.g., 3-anisic acid, vitamin U) to the observed anti-inflammatory or anticancer effects requires further mechanistic investigations, such as rescue experiments or experiments using specific metabolite inhibitors/agonists, for confirmation. KEGG pathway enrichment analysis further elucidated the microbial metabolic basis underlying these metabolite alterations at a systems level. The glycerophospholipid metabolism pathway was significantly enriched in both SN22 and SN05 groups; the marked downregulation of several lysophospholipids (e.g., LPC, LPA) within this pathway suggests strain utilization or transformation of membrane lipid components. SN22-specific enrichment was observed in the thiamine metabolism pathway, associated with coenzyme thiamine pyrophosphate (TPP) synthesis [52]. Enrichment of tyrosine metabolism explained L-dopa generation [53]. Enrichment of the steroid biosynthesis and secondary bile acid biosynthesis pathways indicated SN22’s potential capacity for sterol transformation [54]. Furthermore, enrichment of the amino sugar and nucleotide sugar metabolism pathway corroborated the abundance of CAZymes (particularly GH families) encoded in the SN22 genome, supporting its ability to degrade and utilize complex polysaccharides like insect chitin. In contrast, SN05 enrichment centered on the nitrogen metabolism and alanine, aspartate, and glutamate metabolism pathways, highlighting its active involvement in amino acid utilization and conversion. This provides the metabolic foundation for the enrichment of amino acid-derived metabolites like 5-hydroxytryptophan. These significantly enriched pathways collectively point towards vigorous carbohydrate catabolism, amino acid conversion, energy metabolism, and cofactor/vitamin synthesis activities during LAB fermentation, driving the metabolic profile remodeling. In summary, fermentation-driven metabolic reprogramming not only markedly reduced pro-inflammatory risks but also, through the synergistic enrichment of multiple bioactive metabolites, constitutes the core molecular basis for the subsequent anti-inflammatory, antioxidant, and anti-proliferative effects observed.

This study utilized a DSS-induced mouse model of UC to evaluate the alleviating effects of fermented supernatant on colitis, with a primary focus on whether the fermentation process enhances the anti-inflammatory activity of PA. The results showed that, compared to the non-fermented PA group, the supernatants obtained by fermentation with *L. bulgaricus* SN22 and *S. thermophilus* SN05 alleviated intestinal inflammation to some extent. Serum levels of IL-1β and TNF-α were significantly reduced (*p* < 0.05). Although IL-6 showed a decreasing trend and IL-10 an increasing trend, these changes were not statistically significant (*p* > 0.05). Immunohistochemical results further supported these cytokine trends. HE staining indicated that the fermented group exhibited relatively intact colonic mucosal structure, abundant goblet cells, and milder inflammatory infiltration, crypt destruction, and ulceration. These improvements may be associated with specific metabolites enriched during the fermentation process. Previous studies have reported that fennel extract can alleviate DSS-induced colitis in mice by inhibiting the JAK/STAT1 signaling pathway, restoring the expression and localization of tight junction proteins (such as OCLD and TJP-1), and enhancing intestinal barrier function [55]. Additionally, vitamin U has been shown to activate the Nrf2 antioxidant pathway, promote epithelial proliferation and migration, and contribute to the repair of the DSS-damaged intestinal barrier [49]. Compared with previously reported studies in which DSS-colitis models were treated with fermented products derived from individual strains, the fermented supernatant in this study showed similar improvements in pro-inflammatory cytokine levels and histological scores [56,57]. Moreover, this study is the first to demonstrate that traditional dairy fermentation strains can produce bioactive components with potential anti-inflammatory activity from an insect matrix. Compared to the unfermented PA group, which showed some protective activity, the effect was lower than that of the two fermented groups. This indicates that the enhanced anti-inflammatory activity was not due to the PA matrix itself, but rather to component transformation and enrichment driven by LAB fermentation. A major limitation of this study is the lack of a recognized positive control (such as Sulfasalazine, SASP) and a non-probiotic fermented negative control. Therefore, it is difficult to accurately assess the magnitude of the observed effects or whether they are strain-specific. However, comparing the results of this study with the literature data on the effects of SASP in alleviating DSS-induced UC in mice suggests that the present fermentation strategy may have potential value in mitigating UC-related inflammatory factors [56,58,59]. Second, this study exclusively employed male mice, primarily to minimize potential variability introduced by the estrous cycle on the inflammatory model in this initial exploratory phase. This approach, however, limits the generalizability of the findings across sexes. Additionally, the relatively short intervention period, while suitable for reflecting acute effects, necessitates that the safety and efficacy of long-term intake be evaluated prior to any future translational applications. Based on the findings and limitations of this study, future research should include female animals to enable a more comprehensive assessment. Furthermore, incorporating the control groups mentioned above and conducting more in-depth mechanistic studies are required to elucidate the mode of action of the fermented products. Despite these limitations, this study provides valuable preliminary evidence for understanding how dairy fermentation processes influence the anti-inflammatory activity of insect-derived natural products and lays a foundation for subsequent research in this field.

The persistent presence of intestinal inflammation is a well-established risk factor for the development and progression of colorectal cancer [60], prompting our further evaluation of the potential anti-tumor activity of *L. bulgaricus* SN22 and *S. thermophilus* SN05 fermented supernatants against HT-29 colorectal cancer cells. In vitro results demonstrated that, compared to the control group, both fermented supernatants significantly inhibited the proliferative viability and colony formation ability of HT-29 cells (colony numbers reduced by approximately 28.07% and 43.14% in the SN22 and SN05 groups, respectively). Furthermore, they significantly induced intracellular ROS accumulation and promoted cell apoptosis (TUNEL-positive signal increased approximately 9.27-fold and 13.53-fold in the SN22 and SN05 groups, respectively). This anti-cancer effect is likely primarily attributed to the specific combination of bioactive metabolites enriched during fermentation. We speculate that certain components within this mixture may possess pro-oxidative properties or interfere with the antioxidant defenses of cancer cells, leading to abnormal ROS accumulation. Elevated ROS levels are a well-confirmed key initiating event triggering the mitochondria-dependent apoptotic pathway [61]. This aligns with Wang et al.’s report on specific LAB metabolites inducing HT-29 cell apoptosis via the ROS-mitochondrial axis [62]. Therefore, the significant elevation in ROS levels strongly suggests it is a core molecular mechanism underlying the pro-apoptotic effect mediated by the fermented supernatants. While the primary focus of this study was assessing the UC-alleviating potential of the fermented product, its demonstrated in vitro growth inhibition and potent pro-apoptotic activity against colorectal cancer cells further attest to the broad biological efficacy of the bioactive substances present in the supernatant. It should be noted that the in vitro experiments in this study primarily focused on investigating the potential inhibitory effects of the fermented supernatant on colon cancer cells. Assessing its safety on normal colon epithelial cells (e.g., NCM460 cells) is essential for understanding its therapeutic window, which will constitute a crucial step in subsequent research aimed at elucidating its mechanism of action and translational potential.

## 5. Conclusions

This study comprehensively applied phenotypic analysis, untargeted metabolomics, a DSS-induced colitis mouse model, in vitro HT-29 cell experiments, and whole-genome sequencing to systematically evaluate the metabolic transformation potential and functional effects of PA fermented by *L. bulgaricus* SN22 and *S. thermophilus* SN05. Genomic analysis revealed that both strains are rich in CAZymes, suggesting their capacity for metabolic remodeling of complex insect-based substrates. Metabolomics further indicated that fermentation significantly altered the metabolite profile, enriching metabolites with potential anti-inflammatory, antioxidant, and mucosal repair activities. These changes may collectively contribute to the symptom alleviation observed in the DSS colitis model treated with the fermented supernatant, as well as its effects on inhibiting proliferation and inducing apoptosis in HT-29 cells in vitro. Future studies should further clarify the dose–response relationship of the fermented supernatant and optimize the process to enhance the yield of active components. It will also be essential to isolate and identify the key active metabolites and validate their synergistic mechanisms. Additionally, incorporating positive controls and non-probiotic bacterial negative controls is recommended to strengthen the validity of the conclusions, and exploring whether mixed fermentation yields synergistic effects would be valuable. In summary, this study not only extends the application of traditional dairy fermentation strains to the development of insect-based resources but also deepens the understanding of how LAB generate metabolites through metabolic remodeling that may improve intestinal health and exhibit potential chemopreventive activities.

## Figures and Tables

**Table 1:** Detection of IL-1β, IL-6, TNF-α, and IL-10 positive expression levels (mean ± SD) in colonic tissues of mice by immunohistochemistry.

Groups	IL-1β Positive Tissue	IL-6 Positive Tissue	TNF-α Positive Tissue	IL-10 Positive Tissue
Control	0.3975 ± 0.3902 ^b^	8.7751 ± 1.2382 ^b^	4.5525 ± 0.3785 ^c^	28.4310 ± 4.8476 ^a^
DSS Control	11.2589 ± 4.1045 ^a^	16.1684 ± 1.1437 ^a^	13.9288 ± 0.2243 ^a^	6.0933 ± 1.6426 ^c^
PA	4.6954 ± 2.4160 ^b^	15.1712 ± 2.4183 ^a^	13.8499 ± 0.5609 ^a^	9.8750 ± 2.3059 ^b^
SN05 + PA	1.6956 ± 0.4809 ^b^	2.5385 ± 1.8234 ^c^	4.7360 ± 0.9705 ^c^	16.7861 ± 0.8420 ^b^
SN22 + PA	2.6439 ± 2.6781 ^b^	14.5638 ± 1.2164 ^a^	6.7198 ± 1.4300 ^b^	14.0640 ± 3.3898 ^c^

Different lowercase letters within the same column indicate significant differences (*p* < 0.05).

**Table 2:** Genomic features of *L. bulgaricus* SN22 and *S. thermophilus* SN05.

Attributes	SN22	SN05
Reads Number	152,407	72,574
Base Number (bp)	1,198,293,806	495,867,121
Mean Length (bp)	7862.46	6832.57
N50 (bp)	8820	7669
Polished Contigs	1	1
Max Contig Length (bp)	1,775,614	1,945,731
N50 Contig Length (bp)	1,775,614	1,945,731
Sum of Contig Lengths (bp)	1,775,614	1,945,731
GC Count (%)	50.05	39.06
Coding Gene Number	1786	2034
Coding Gene Average Length (bp)	835.29	798.23

## Data Availability

The raw data of the genomes have been submitted to the China National GeneBank DataBase (https://db.cngb.org/, accessed on 1 January 2026) with the accession number CNP0007498. Queries may be addressed to the contact authors.

## Supplementary Materials

The following supporting information can be downloaded at: https://www.mdpi.com/article/10.3390/microorganisms14020301/s1. Supplementary Figure S1. ANI visualization of *L. bulgaricus* SN22 and *S. thermophilus* SN05. (A) ANI values of *L. bulgaricus* SN22 with other conspecific strains. (B) ANI values of *S. thermophilus* SN05 with other conspecific strains. Supplementary Figure S2. Phylogenetic tree and functional annotation of the *S. thermophilus* SN05 genome. (A) Phylogenetic tree of SN05 and 14 conspecific strains based on core genome sequences. (B) Circular genomic map depicting gene locations and annotation results for the SN05 genome. (C–E) Functional annotation of the SN05 genome using NCBI Blast+: KEGG pathways (C), COG categories (D), and GO terms (E).
