# Supplementary material for: Alleviation of Ulcerative Colitis in Mice by Individual Fermentation of Periplaneta americana Powder with L. bulgaricus SN22 and S. thermophilus SN05
Source: Microorganisms. 2026 Jan 27;14(2):301. doi: 10.3390/microorganisms14020301 (PMC12943602; doi:10.3390/microorganisms14020301)
Supplement: Supplementary file 1 [file microorganisms-14-00301-s001.zip › microorganisms-4078231-supplementary.pdf]

Supplementary Material

1 Supplementary Figures

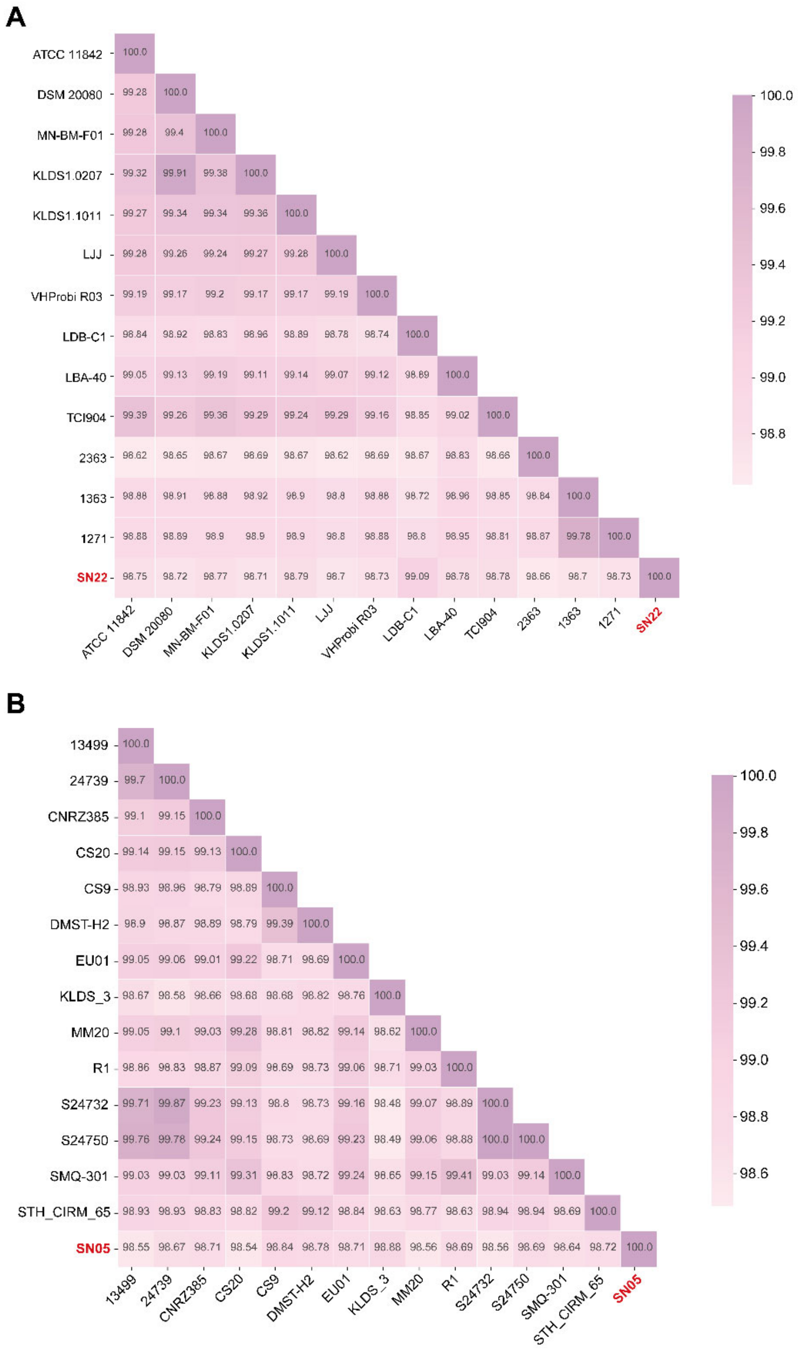

Supplementary Figure S1. ANI visualization of *L. bulgaricus* SN22 and *S. thermophilus* SN05. (A) ANI values of *L. bulgaricus* SN22 with other conspecific strains. **(B)** ANI values of *S. thermophilus* SN05 with other conspecific strains.

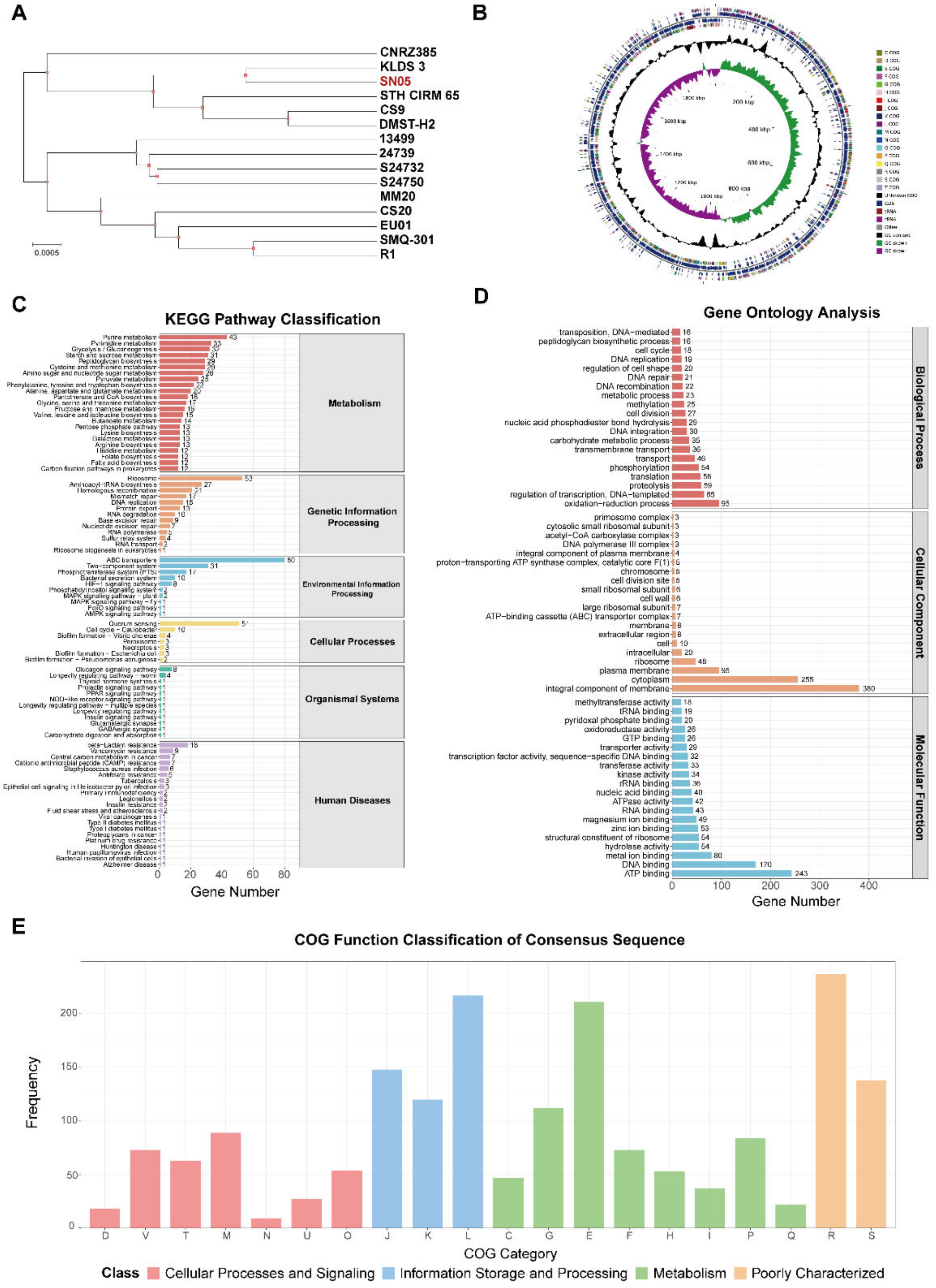

Supplementary Figure S2. Phylogenetic tree and functional annotation of the *S. thermophilus* SN05 genome. (A) Phylogenetic tree of SN05 and 14 conspecific strains based on core genome sequences. **(B)** Circular genomic map depicting gene locations and annotation results for the SN05 genome. **(C-E)** Functional annotation of the SN05 genome using NCBI Blast+: KEGG pathways (C), COG categories (D), and GO terms (E).
